# Simulating the blood transfusion system in Kenya: Modelling methods and exploratory analyses

**DOI:** 10.1371/journal.pgph.0004587

**Published:** 2025-08-13

**Authors:** Yiqi Tian, Bo Zeng, Jana MacLeod, Gatwiri Murithi, Cindy M. Makanga, Hillary Barmasai, Linda S. Barnes, Rahul S. Bidanda, Tecla Chelagat, Tonny Ejilkon Epuu, Abdirahaman Musa, Robert Kamu Kaburu, Jason Madan, Jennifer Makin, Alejandro Munoz-Valencia, Carolyne Njoki, Kevin Ochieng, Bernard Oduor Olayo, Jose Ricardo Paiz, Kristina E. Rudd, Mark Yazer, Juan Carlos Puyana, Bopaya Bidanda, Jayant Rajgopal, Pratap Kumar

**Affiliations:** 1 Department of Industrial Engineering, University of Pittsburgh, Pittsburgh, Pennsylvania, United States of America; 2 Kenyatta University College of Health Sciences School of Medicine, Nairobi, Kenya; 3 Institute of Healthcare Management, Strathmore University Business School, Nairobi, Kenya; 4 Center for Public Health and Development, Kisumu, Kenya; 5 Kenya Blood Transfusion and Transplant Service, Nairobi, Kenya; 6 Linda S. Barnes Consulting, Seattle, Washington, United States of America; 7 Ministry of Health & Sanitation, Turkana County Government, Turkana, Kenya; 8 Warwick Medical School, University of Warwick, Coventry, United Kingdom; 9 Department of Obstetrics, Gynecology, and Reproductive Sciences, University of Pittsburgh Medical Center Magee Women’s Hospital, Pittsburgh, Pennsylvania, United States of America; 10 Department of Anesthesiology and Perioperative Medicine, University of Pittsburgh, Pittsburgh, Pennsylvania, United States of America; 11 Department of Anesthesia, Aga Khan University Nairobi, Nairobi, Kenya; 12 Surgery Department, Egerton University, Egerton, Kenya; 13 Department of Surgery, University of Pittsburgh, Pittsburgh, Pennsylvania, United States of America; 14 Department of Critical Care Medicine, Center for Research, Investigation, and Systems Modeling of Acute Illness (CRISMA), University of Pittsburgh, Pittsburgh, Pennsylvania, United States of America; 15 Department of Pathology, University of Pittsburgh, Pittsburgh, Pennsylvania, United States of America; 16 Departments of Surgery and Critical Care Medicine, University of Pittsburgh, Pittsburgh, Pennsylvania, United States of America; 17 Department of Global Surgery, Royal College of Surgeons in Ireland, Dublin, Ireland; 18 Global Business School for Health, UCL, London, United Kingdom; McGill University, CANADA

## Abstract

The process of collecting blood from donors and making it available for transfusion requires a complex series of operations involving multiple actors and different resources at each step. Ensuring hospitals receive adequate and safe blood for transfusion is a common challenge across low- and middle-income countries, but is rarely addressed from a system level. This paper presents the first use of discrete event simulation to study the blood system in Kenya and to explore the effect of variations and perturbations at different steps of the system on meeting blood demand at patient level. A process map of the Kenyan blood system was developed to capture critical steps from blood donation to transfusion using interviews with blood bank, hospital and laboratory personnel at four public hospitals across three counties in Kenya. The blood system was simulated starting with blood collection, a blood bank where blood is tested and stored before it is issued, a major hospital attached to the blood bank, and several smaller hospitals served by the same blood bank. Values for supply-side parameters were based mainly on expert opinion; demand-side parameters were based on data from blood requisitions made in hospital wards, and dispatch of blood from the hospital laboratory. Illustrative examples demonstrate how the model can be used to explore impacts of changes in blood collection (e.g., prioritising different donor types), blood demand (e.g., differing clinical case mix), and blood distribution (e.g., restocking strategies) on meeting demand at patient level. The model can reveal potential process impediments in the blood system and aid in choosing between alternate strategies or policies for improving blood collection, testing, distribution or use. Such a systems approach allows for interventions at different steps in the blood continuum to be tested on blood availability for different patients presenting at diverse hospitals across the country.

## Introduction

Blood and blood products are critical to improving patient outcomes across a wide range of medical situations from emergencies to chronic illnesses. Maintaining a stable and sustainable supply of blood products entails a complex series of events within the vein-to-vein system, beginning with blood donation and ending with blood transfusion. Variations of these events are dictated by the availability of blood donors, supply chain and facilities limitations, and regional or national regulations and customs. Given the multitude of places where interventions could be introduced to make improvements in the process, a holistic, high-level perspective of the entire system can be very helpful when attempting to streamline the efficiency of the blood donation and transfusion processes [1,2].

Such a perspective is especially important in resource-constrained health systems where the mismatch between supply and demand is most pronounced [3]. The World Health Organization (WHO) reports that African countries annually collect only 5% of the global donated blood supply, despite constituting 14% of the world’s population [4]. The WHO estimates that collection rates are as high as 53.0 units per 1,000 population in high-income countries; Kenya however is estimated to collect less than 10 units per 1,000 population annually, although recent increases in donation rates are reported [4,5]. This paucity is further accentuated by a high prevalence of conditions where blood product administration is an integral component of clinical management. Sickle cell disease, malaria, gastrointestinal parasite infestation such as hookworm, and nutritional deficiencies are prevalent and often associated with clinically significant anaemia requiring blood transfusion, especially in children [6,7]. Two out of five maternal deaths in Kenya are related to obstetric haemorrhage [8–11]; obstetric emergencies and trauma patients are disproportionately affected by blood supply deficiencies, as well as time factors because of limited infrastructure and poor referral networks [12,13].

In Kenya, blood products for transfusion are collected and regulated by the Kenya Blood Transfusion and Transplant Service (KBTTS). The KBTTS structure consists of a national coordinating unit and six Regional Blood Transfusion centres (RBTCs) that collect, test, store, and distribute blood products, with an additional 43 satellite blood centres that collect blood but send samples to RBTC for testing [14]. The transfusion system has undergone significant recent shocks, starting with funding shortfalls [15], followed shortly thereafter by the COVID-19 pandemic that resulted in long-term school closures and therefore significant drop in blood collection (blood donation drives in Kenya and much of Sub-Saharan Africa target high school students aged 16 years and older) [16–18]. The Pathways for Innovation in Blood Transfusion Services in Kenya (PITS Kenya) study, funded through a BLOODSAFE program grant from the National Heart, Lung, and Blood Institute (NHLBI), aims to understand the blood system in Kenya and implement innovations to improve blood availability at the point-of-care [19]. The study focuses on three distinct counties in Kenya - Turkana, Siaya and Nakuru. The counties were chosen to represent diverse challenges facing access to safe blood for transfusion in Kenya including remote geographies, socio-economic disparities, cultural beliefs, disease prevalence and healthcare infrastructure.

Discrete event simulation (DES) is a computer-based modeling approach that tracks how a system evolves over time in a stochastic environment. DES models are characterised by discrete events that occur in continuous time. The events involve entities progressing through pre-specified processes that have their individual features such as times, capacities and resource requirements. DES models have been used to analyse and optimise the design and/or operation of systems in a wide range of domains, and have been recognized as a flexible and powerful tool for predicting and quantifying the response of systems to change [20]. These changes are typically in the form of planned interventions and with a simulation model one can analyse the effects of these interventions before making any changes in the real world [21]. Examples of application areas in healthcare include nurse scheduling [22], emergency department operations [23], and strategizing vaccine distribution processes [24]. A recent review identified 231 studies focused on DES modelling in healthcare. The majority of these were conducted in high-resource settings; few studies applied DES to LMIC healthcare settings, and none tackled the challenges of blood supply in LMICs [25].

This paper describes the design and construction of a DES model for the entire blood continuum in Kenya, spanning collection, storage, delivery and utilization. It uses data from three representative counties, and aims to help identify and better understand which elements of the system its performance is especially sensitive to, and to also quantify the effect of planned interventions to improve system performance. Using illustrative examples, the paper demonstrates the potential of a simulation model to assist in understanding the obstacles in the blood continuum, and to guide effective policy or strategy decisions.

## Materials and methods

From 01 July 2022–24 March 2023, a mixed-methods approach was employed to acquire data on the blood continuum in four locations (large hospitals which routinely collected blood from donors) within three Kenyan counties: Siaya, Turkana, and Nakuru (Nakuru County included two locations). The methods for the broader PITS Kenya research program have been previously published [19]. The three counties were purposively selected to represent diverse socioeconomic and epidemiological settings for healthcare delivery across Kenya.

### Ethics statement

This study protocol was reviewed and approved by the Strathmore University Institutional Ethic Review Committee (reference number #SU-IERC0992/21, #SU-IERC1345/22), the Kenyan National Commission for Science, Technology & Innovation (license number NACOSTI/P/21/9976, NACOSTI/P/22/17897), and the University of Pittsburgh Institutional Review Board (reference number #STUDY21020040). Interviews with blood system stakeholders were conducted between 5 August 2021 and 19 April 2022. Informed consent was obtained in writing from each participant category – patient, donor, clinician, blood bank administrator, health facility administrator, and health system administrator. The protocol, framework and methods for the wider study are described in a previous publication [19]. Additional information regarding the ethical, cultural, and scientific considerations specific to inclusivity in global research is included in S1 Checklist.

### Process mapping

The first step in the development of the DES model was a process mapping exercise, with the goal of understanding and describing the different processes in the blood system, their interrelationships, and the overall workflow. This allowed us to identify key elements that describe the system that formed the basis for the DES model. A process map is a visual representation of workflow within a system that comprises different processes aimed at delivering a good or service. In the context of the blood continuum in Kenya that we are addressing, the service provided is the collection and delivery of blood, and the process map captures the steps/processes that takes place in the provision of this service, starting with blood collection and ending with delivery of blood to the clinicians who request it. Broadly speaking the process has three stages: (1) collection, (2) processing and storage, and (3) delivery and use.

Development of process maps of large, distributed systems is typically time consuming, laborious, and iterative. The wider qualitative study engaged a diverse group of stakeholders including laboratory personnel, blood bank directors, clinicians (consultants, medical officers, and clinical officers), nurses, blood donors, and patients across four different locations [19]. The detailed methods of qualitative data collection and analysis, which are not limited to process mapping of the blood system, will be described in a separate manuscript. The engineering team developed first versions of the process map at four blood banks across three counties based on meetings with study team members based in each of these sites. Draft maps were presented to a collection of experts for feedback [26], especially hospital laboratory and blood bank personnel at each location (blood banks were located within the hospital premises in the four locations). These experts contributed and corroborated detailed information on local operational practices including testing procedures, processing times, and test turnaround metrics. Expert feedback was systematically documented, with discrepancies discussed and resolved, and maps refined further to capture both common practices and regional variations. The second version of the process map was presented to a different set of national and county blood system stakeholders at a central stakeholder workshop in Nairobi (part of broader stakeholder engagement of the PITS Kenya study). These participants further validated the steps in the processes map and the initial design of the DES model.

The final process map was generated (S1 Fig) to capture the three broad stages of the blood continuum, and also revealed both commonalities and differences across the four locations (represented by colour codes). Commonalities include the sources of blood, which in all locations were blood drives, replacement donors and voluntary (drop-in) donors. Differences were observed, for example, in where blood was tested for transfusion-transmissible infections (TTIs); one location conducted on-site testing, while the other three sent blood samples to RBTCs for TTI testing.

### Kenya blood system model

As suggested recently by Jacobs et al. [2] this study takes a holistic approach to build a comprehensive model that captures the primary features of the entire blood continuum in Kenya. The primary elements of the model include blood collection processes and facilities (satellite collection centres or RBTCs) in the collection stage; transportation, testing, componentization and storage of blood in the processing stage; and distribution to hospital laboratories, clinical practices and patient types (e.g., emergency or non-emergency) in the delivery and use stage.

There are too many elements to be able to model and simulate every detail and variation of processes in the blood continuum. To rationalise our large-scale system model, some simplifications were made in the form of combining steps where adding more detail offers little benefit or where we do not have sufficient data to be able to add the level of detail that we would like. For example, we did not include the detailed sequence of steps and times in the blood collection process at a donation centre (e.g., donor screening using questionnaires and measurements of weight and haemoglobin), details of laboratory equipment and processes used for TTI testing, or details on specific blood storage devices at different locations. Moreover, we did not explicitly model the demand and use of different blood products such as whole blood, plasma, platelets, etc. Similarly, while objective human behaviour has been captured and included in the model – such as how frequently blood drives are conducted in the community, or how often donor blood samples are batched and sent for TTI testing – capturing subjective human behaviour (e.g., hesitancy to donate or accept blood transfusions based on personal or cultural beliefs) is beyond the scope of the current model. Processes common across the study locations were prioritised in order to build a generic model which could be readily adapted to the different locations. This yielded a framework for a model that would be a reasonable representation of the system with sufficient details on the critical components, and at the same time, be feasible to simulate and analyse. As described above for the process map, the representation of the Kenyan blood system in the DES model was presented for validation to national and county blood system stakeholders at a central stakeholder workshop in Nairobi.

**Fig 1:**
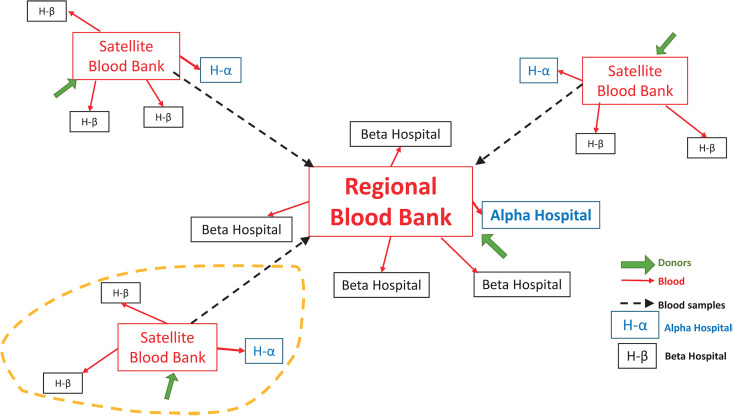
Blood collection and delivery subsystems.

This model framework consists of a blood collection centre (satellite or RBTC) that is located within or alongside a large hospital (“Alpha hospital”), and a set of smaller, geographically distant hospitals (“Beta hospitals”), that depend on the same collection centre for their blood supply. This framework, illustrated in Fig 1, using one RBTC and three satellite collection centres and the Alpha and Beta hospitals they serve, represents a modular subsystem that is repeated to form the national blood system; the simulation in this study was conducted on a smaller unit highlighted by a yellow circle, comprising of one satellite blood collection centre, one Alpha hospital and three Beta hospitals. The different stages of the blood continuum are modelled as follows:

#### Blood collection.

Blood is collected at collection centres using three different modes: blood drives, family replacement donors (FRD), and walk-in/volunteer donors. Blood drives are organised by the collection centre to take place at two broad types of locations: (a) institutions such as schools or military units, or (b) locations within the community such as town-centres or religious sites. FRD are relatives or friends of a patient requiring a blood transfusion, who are mobilised to donate by the patient or their caregivers. Volunteer donors are individuals presenting to the collection centre to give an undirected donation (not in response to any specific patient’s needs). All donors are administered a health questionnaire followed by a preliminary physical screening to ensure their eligibility to donate blood as per the KBTTS guidelines. For various reasons such as risk factors or pre-existing conditions, not all donors who present to donate blood will pass this preliminary screening.

#### Blood processing and storage.

Collected blood is tested for TTIs before it can be qualified for use and stored. The model does not currently involve componentization of blood units as this paper focuses on the use of whole blood. Samples from the satellite centres are sent to the nearest RBTC for testing, but only on specific days of the week to ensure there is a sufficient number of samples in each batch transported. Any TTI-positive blood is discarded, and only the qualified blood is stored at the collection centre. Collection centres have a finite amount of storage capacity for blood, both pre-qualified and qualified.

#### Blood delivery and use.

**Fig 2:**
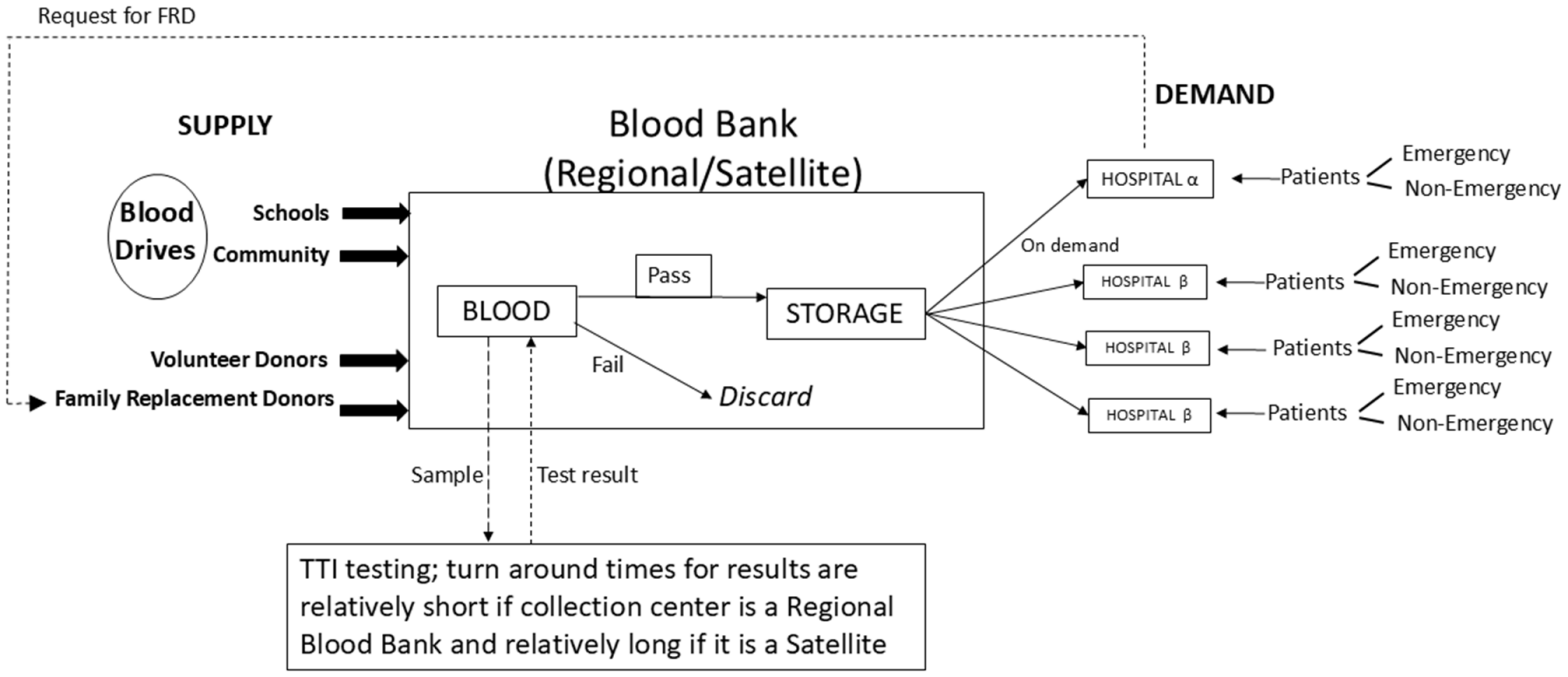
Blood subsystem operations.

The demand side of the model explores how blood is consumed at hospitals. Daily demand for blood is generated at each hospital by patients who fall into one of two categories based on timing of need: emergency and non-emergency. When this clinical need occurs, a request is sent to the blood collection centre where qualified blood is stored (this reflects current processes across all locations studied). The amount of time that elapses before the blood reaches the patient depends on the hospital setting; this is relatively short if it is an Alpha hospital (which is typically close to the collection centre), and longer if it is a Beta hospital (which is usually further away from a collection centre). Fig 2 depicts the operations of this subsystem schematically.

### Discrete event simulation (DES)

The Simio software was used to perform the DES modelling in this study [27]. The DES model was used to mimic the behaviour of the blood continuum over a 12-month study period. The system performance measure selected for this study was the percentage of patients who have their blood demand satisfied over the one-year period. Other metrics such as time to satisfy blood demand will be explored in future iterations.

### Simulation inputs and sources

The values of the input parameters used in the simulation were obtained from various sources ranging from recorded data and observations, to estimates and opinions from those with domain expertise as described above. Tables 1 and 2 detail the input data used in the simulation for the blood collection & processing, and distribution & use stages of the blood continuum, respectively.

**Table 1:** Blood collection and processing parameters.

	Parameter	Probability Distribution	Source
COLLECTION			
Institutional Drives	every odd month; mean = 100/session	Poisson; *λ* = 100	Blood drive records, blood bank staff
Community Drives	every even month; mean = 30/session	Poisson; *λ* = 30	
Walk-In donors	Mon., Wed., Fri.: mean = 1/day Tue., Thu. mean = 0.5/day	Poisson; *λ* = 1 Poisson; *λ* = 0.5	
FRD response rates			
Donors per Alpha hospital patient:	0 = 65%; 1 = 25%; 2 = 10%	Discrete; *p*(0,1,2)= (0.65,0.25,0.1)	Blood bank staff, Clinical staff
Donors per Beta hospital patient:	0 = 90%; 1 = 10%	Discrete; *p*(0,1)=(0.9,0.1)	
ELIGIBILITY TESTING			
Donor pass rate (FRD)	80%	Bernoulli; *p* = 0.8	Blood bank staff
Donor pass rate (other)	90%	Bernoulli; *p* = 0.9	
TTI SCREENING			
Sample transport time (hours)	Every Tue., Thu. Satellite = 5; RBTC = 1	–	Blood bank staff
Turn-around time estimates (hours)	low = 4; likely = 12; high = 24	Triangular (*a* = 4, *m* = 12, *b* = 24)	
TTI test pass rate	FRD = 90%; others = 98%	Bernoulli; *p* = 0.9 or *p* = 0.98	

**Table 2:** Blood distribution and use parameters.

	Parameter	Probability Distribution	Source
FOR ALPHA HOSPITALS			
Emergency patients	average of 1.3 per day	Poisson; *λ* = 1.3	Blood requisition records, clinical staff
Non-emergency patients	average of 4.7 per day	Poisson; *λ* = 4.7	
Blood units per patient	1 = 35%; 2 = 50%; 3 = 15%	Discrete; *p*(1,2,3)= (0.35,0.50,0.15)	
Transportation time estimates (hours)	low = 1; likely = 3; high = 12	Triangular (*a* = 1, *m* = 3, *b* = 12)	Blood bank staff
Maximum emergency patient wait time (hours)	low = 4; likely = 24; high = 36	Triangular (*a* = 4, *m* = 24, *b* = 36)	Clinical staff
Maximum non-emergency patient wait time (hours)	low = 48; likely = 72; high = 168	Triangular (*a* = 48, *m* = 72, *b* = 168)	
FOR BETA HOSPITALS			
Emergency patients	30% of Alpha hospital rate	Poisson; *λ* = 0.39	Clinical staff
Non-emergency patients	30% of Alpha hospital rate	Poisson; *λ* = 1.441	
Blood units per patient	1 = 35%; 2 = 50%; 3 = 15%	Discrete; p(1,2,3)=(0.35,0.50,0.15)	
Transportation time estimates (hours)	low = 12; likely = 48; high = 96	Triangular (*a* = 12,*m* = 48, *b* = 96)	Laboratory staff
Maximum emergency patient wait time (hours)	low = 4; likely = 24; high = 36	Triangular (*a* = 4, *m* = 24, *b* = 36)	Clinical staff
Maximum non-emergency patient wait time (hours)	low = 48; likely = 72; high = 168	Triangular (*a* = 48, *m* = 72, *b* = 168)	

The simulation assumes that random arrivals of blood donors and patients are according to a Poisson process. Estimates of times that are random (transportation times, patient wait times) were based on a triangular distribution with three consensus estimates provided by experts: low, most likely and high. Binary random variables (test pass rates) were modelled with a Binomial distribution. Lastly, some random variables that could take on multiple discrete values (e.g., blood units per patient, FRD response rates) were modelled using discrete probabilities.

Data for average number of donors at blood drives and average number of family replacement donors mobilised per patient are based on review of blood drive records and interviews with blood bank and clinical staff in the study locations (Table 1). Patient blood need at an Alpha hospital is based on quantitative data from blood requisition records as described in a previous publication [19]; similar demand data was not collected at Beta hospitals for this study, but is part of ongoing work. Thus, in the simulation, the patient mix at a Beta hospital was assumed to be similar to an Alpha hospital but reduced to 30% of the value. Estimates of the range of maximum patient wait times for blood (for both emergency and non-emergency patients) can vary depending on the patient’s specific condition, and therefore, the estimates used in the model (Table 2) were obtained in consultation with local study site clinicians. Wait times refer to the length of time a patient remains in the simulation needing blood (e.g., patients may die or be discharged if their blood need is not met in time).

### Simulation outputs

A patient-centric measure was selected in this study to evaluate blood system performance: the percentage of patients who have their blood demand met (either partially or fully). While the simulation can differentiate between fully and partially met (only some of the blood units requested are dispatched) demand, the results described here combine both into one measure of met demand. A three-month warm-up period was used in the simulation to allow the system to reach a steady state and to overcome any bias from the initial starting state of the system; all output data for this initial interval of time was discarded. The outputs for the following 12-month period were used to evaluate the system’s performance. The simulation is repeated 100 times, and the outputs generated from each run are recorded for further analysis. Given the inherent variability in the system, the performance measure (percentage of met demand over a one-year period) from one run to another will vary. Note that a specific simulation run models the occurrence of random events (e.g., the generation of blood demand at a hospital, the specific mix of emergency vs. non-emergency patients and their individual wait times, the donation of blood at a donation centre and its qualification/disqualification, etc.). The specific realizations of these events are based on the underlying probability distributions of the input parameters. While these distributions and parameters remain unchanged from run to run, their actual realizations will be distinct (albeit “similar”) in each simulation run. When we look at the system’s performance across a large number of runs (100 in our case), it gives us a good idea of its overall performance. Typically, we would construct a confidence interval around the mean to see what the range of performance might be, but our approach does not capture second order uncertainty, i.e., uncertainty about values of the parameters that we have used. Consequently, we employ a box-and-whisker plot to display the system’s performance. This visualization emphasizes the median as well as the upper and lower quartiles, thereby offering a depiction of the variability across the simulation runs.

## Results

Three experiments were chosen to illustrate the utility of the model. These examples were specifically chosen to illustrate the overarching “vein-to-vein” nature of the model, with experiments addressing the three different stages of the blood continuum: blood collection, processing & storage, and distribution & use.

### Experiment 1

The first experiment simulates the impact of increasing blood supply in the system on meeting blood demand. Increased supply was simulated in two ways by increasing by roughly 50%, relative to baseline, either a) the total number of annual blood drives, or b) the number of FRDs mobilised per patient. The two changes represent two different ways in which blood supply is mobilised: the first is relatively unlinked to demand (blood drives are scheduled in advance and mostly conducted independent of hospital need for blood), while the second is directly linked to patients needing blood. In the simulation the changes were implemented by a) increasing the number of community drives by holding them once every month rather than once every other month; annual blood drives increased from 12 (6 institutional + 6 community; Table 1) to 18 (6 institutional + 12 community), or b) increasing FRD rates from p(0,1,2)=(65%,25%,10%) to p(0,1,2)=(45%,40%,15%) at the Alpha hospital, and from p(0,1)=(90%,1%) to p(0,1)=(85%,15%) at Beta hospitals.

**Fig 3:**
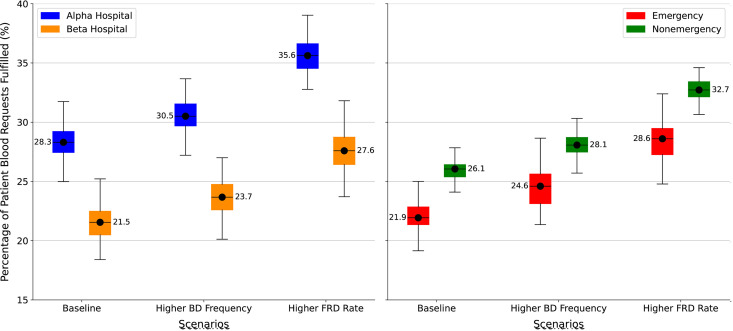
Increased supply: met demand for blood by hospital type (left) and patient type (right). Abbreviations: BD, Blood Drive; FRD, Family Replacement Donor.

Fig 3 (left) displays, across the 100 runs, the percentage of patients whose blood order requests are fulfilled at baseline, and for each of the two simulation cases noted above; these are presented separately for the Alpha and Beta hospitals. As the figure shows, across all scenarios, Beta hospitals have a median met demand that is roughly 7–8 percentage points lower than the Alpha hospital: 21.5% (20.5-22.5%) vs 28.3% (27.4-29.2%). This is presumably because they are further away from the blood bank. For both Alpha and Beta hospitals there is a clear improvement in performance over the baseline with either scenario for increasing blood supply. This improvement in meeting demand is however much more pronounced with the increased blood supply from FRDs as compared to the increase from community blood drives: 35.6% vs 30.5% in Alpha and 27.6% vs 23.7% for beta hospitals, respectively.

Fig 3 (right) also displays the results of Experiment 1, displayed by patient type (emergency vs. non-emergency). At baseline, non-emergency patients have a higher median rate of met demand compared to emergency patients (26.1%; IQR 25.4-26.4%) vs 21.9%; IQR 21.3-22.9%, respectively), likely because they can wait longer to receive blood. When the number of community blood drives is doubled, fulfilment of blood requests rises by approx. 2% for each patient type, to 28.1% (27.5-28.7%) for non-emergency and 24.6% (23.1-25.6%) for emergency patients. However, with doubling FRD rates, the outcome improves by nearly 7% for both patient types, to 32.7% (32.1-33.4%) for non-emergency patients and 28.6% (27.3-29.5%) for emergency patients.

### Experiment 2

The second experiment focuses on the demand side for blood, and explores the change in met demand when the patient case mix (i.e., proportion of emergency vs. non-emergency patients, or E/NE ratio) changes from the baseline values. Although the patient mix is not something that can be directly controlled, the results of this experiment could provide guidance on anticipated blood need at different hospital locations depending upon local case mix. For the same average load of 6 patients per day at an Alpha hospital, the daily average E/NE ratio is changed from a baseline of 1.3/4.7 (Table 1) to 2.3/3.7. A contrasting scenario (E/NE ratio of 0.3/5.7) where there is a significantly larger share of non-emergency patients than in the baseline, was also studied. It was assumed the demand case mix for each Beta hospital is the same on average as at the Alpha hospital. A third scenario was also simulated where the patient mix is unchanged from the baseline, but some event (e.g., a road traffic accident with multiple severely injured patients) causes a sudden spike in the number of emergency patients at every hospital once per month.

**Fig 4:**
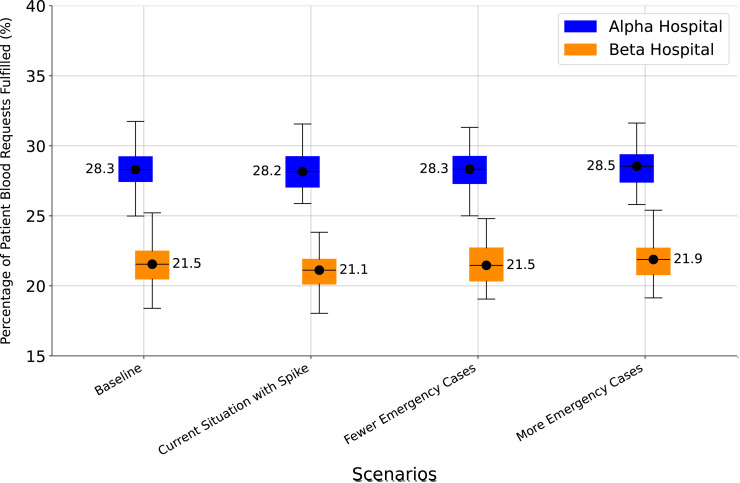
Change in demand mix: met demand for blood by hospital type.

Fig 4 displays the percentage of patients whose blood demand is satisfied, for the baseline and for each of the above three scenarios, and disaggregated between Alpha and Beta hospitals. The overall baseline pattern of improved performance at Alpha hospitals continues to be shown in all three scenarios. In each of the three scenarios there is no difference in median met demand relative to baseline. Therefore, assuming other model inputs and assumptions are constant, change in the proportion of patients with emergency and non-emergency blood needs does not appear to have a significant impact on the system.

### Experiment 3

The third experiment examines the effects of operational and policy changes in how blood is distributed to and used in hospitals. The baseline model captures current operations of the Kenyan blood system where there is no minimum stock level maintained as a policy, and all blood is delivered to the hospital from the blood bank based on patient need. Given the time required to transport blood to the hospital from the blood bank, this could delay availability of blood, especially for patients who need blood emergently, and especially at a Beta hospital that is relatively distant from the blood bank serving it. Therefore, an alternative scenario was simulated in which periodic automatic replacement (PAR) is implemented at each hospital. In this scenario, a small inventory of blood (PAR amount) is maintained at the hospital. In this simulation, five units of tested blood are supplied from the blood bank at the beginning of every week irrespective of hospital inventory. This blood is placed into storage at the hospital and made available to patients on a first-come, first-served basis. Two different restocking scenarios were explored, one where the PAR amounts were maintained only at Beta hospitals (Refill (Beta Only)), which are distant from blood banks, and another where PAR amounts are maintained at both Alpha and Beta hospitals (Refill (Both)).

**Fig 5:**
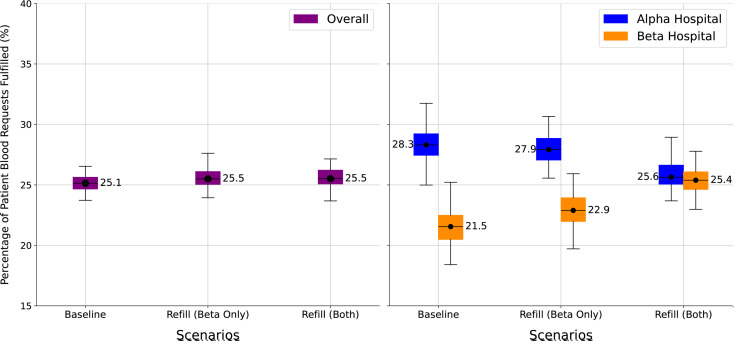
Changes in restocking policies: overall met demand for blood (left) and met demand by hospital type (right).

Fig 5 (left) shows that the system performance is virtually unchanged with both strategies, with no appreciable difference between them from baseline. However, the results in Fig 5 (right), where the performance is separated out by type of hospital, are different and provide more insight. With the Refill (Beta only) strategy, the drop in performance at the Alpha hospital is minimal (27.9%; 27.1-28.8%), but with modest improvement in performance at the Beta hospitals (22.9%; 22.0-23.9%). With the Refill (Both) strategy, while met demand at Beta hospitals improves by about 4%, from 21.5% (20.5-22.5%) at baseline to 25.4% (24.6-26.1%). This outcome illustrates a rather complex interplay between blood requests arising from restocking policies and patient need. In a paradigm of supply shortage, the Alpha hospital could perform worse because a portion of the blood supply from blood banks is diverted to restocking Beta hospitals, and patient-driven requests from Alpha hospitals may come after similar requests from Beta hospitals (the model uses a first-come, first-served approach to supply hospitals).

**Fig 6:**
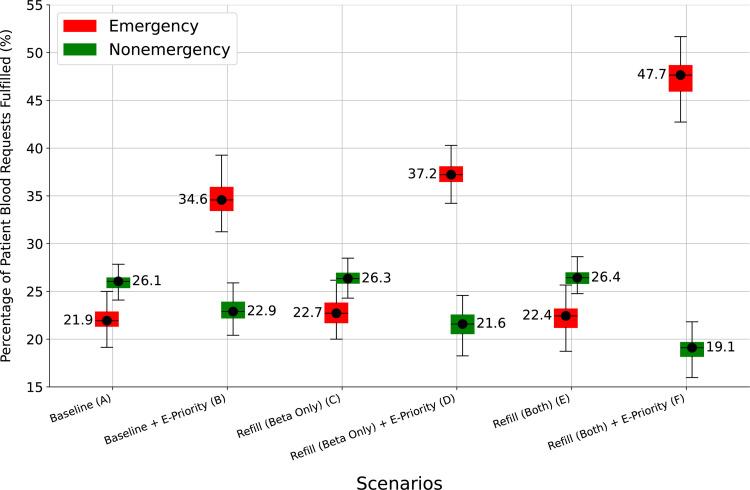
Restocking policies: met demand by patient type. Abbreviations: E-Only, blood prioritised for emergency patients only.

An additional scenario was developed with a variation in the blood administration policy, where any blood in hospital storage (e.g., blood units that were delivered to the hospital but not transfused, possibly because the patient for whom blood was requested died before transfusion) is reserved only for emergency patients (E-priority). Fig 6 shows results for the two different baselines (scenarios A, B), one with the current first-come first-served policy (met demand for non-emergency patients 26.1% (25.4-26.6%) vs. 21.9% (21.3-22.9%) for emergency patients) and one with priority for emergency patients (met demand for non-emergency patients 22.9% (22.2-23.9%) vs. 34.6% (33.5-35.9%) for emergency patients). The E-priority policy, reserving any stored blood for emergency patients improves met demand for emergency patients by about 13%, but met demand for non-emergency patients decreases by about 3% (scenario B). However, there is no difference in overall met demand for blood between the two different policies (around 25% in both cases), presumably because the number of emergency patients is only about a quarter of the number of non-emergency patients.

There is no impact on performance with a PAR strategy for either type of patient with the current first-come, first-served policy for access to stored blood (scenarios A, C, and E in Fig 6). However, implementing the E-priority policy alongside a PAR strategy leads to additional increases in met demand for emergency patients (scenarios B, D and F in Fig 6), with varying reductions in met demand for non-emergency patients. In particular, the largest increase in meeting blood demand for emergency patients is with PAR at both Alpha and Beta hospitals as opposed to PAR at just the Beta hospitals (scenario F). In patient numbers, the baseline with E-priority policy (scenario B) met blood demand for 312 emergency patients and 751 non-emergency patients (total of 1063 patients) in the simulation, on average over one year. When the E-priority policy is augmented with PAR at both hospital types (Scenario F), there are on average, 429 emergency and 627 non-emergency patients with met demand (total of 1056 patients). In other words, although the total number of patients with met demand is almost the same, there are on average 117 additional emergency patients who receive (potentially lifesaving) blood, at the expense of 124 fewer non-emergency patients who might be compromised by not receiving the blood they need.

## Discussion

This novel study presents a comprehensive DES model of the blood transfusion system in Kenya, developed from data and insights across three diverse Kenyan counties, and demonstrates its usefulness in evaluating potential initiatives to improve the availability of safe blood for transfusion. This is the first attempt to simulate the entire blood supply chain from donation to transfusion at patient level in resource-constrained settings typical of most low- and middle-income country (LMIC) contexts. This comprehensive simulation model highlights the complexities and interdependencies of various stages within the blood transfusion continuum, and could provide policy makers with an analytic tool to assess various interventions in the blood system, helping to optimize blood availability for patients with conditions ranging from maternal emergencies to chronic diseases and cancer.

The few previous DES studies on blood systems in high-resource settings have typically focused on individual segments of the transfusion chain, such as the use of simulation to improve human resource allocation and management in blood collection systems [28], to optimise operational efficiency and service quality within a collection centre [29], or to address inventory strategies in blood centres, exploring ways to minimise wastage and shortage [30]. However, these prior studies do not encompass the complete blood transfusion process, including hospital processes, patient needs, and the potential interaction between patients and donors. While these studies provide valuable insights into parts of the overall process, they overlook the broader system and unique aspects of blood systems in LMICs like reliance on FRDs, limited testing capacity, diverse case mix driving blood demand, and other local socioeconomic, political, financial and clinical constraints affecting blood transfusion.

The DES model presented here offers a novel, unifying framework that captures key aspects across the entire blood transfusion continuum from donor accrual to patient transfusion. Each step within the system, from blood drives and donor eligibility to testing, storage, and distribution, involves numerous complexities and potential bottlenecks. By simulating these interconnected processes, the model can reveal critical points where interventions can yield the most significant improvements, offering a powerful tool to optimise the entire blood supply chain. One of the key features in this model is the differentiation between Alpha and Beta hospitals based on their proximity to blood banks. There are 49 blood collection centres in Kenya which collect and store blood from donors (of which six RBTCs also test blood for TTIs) [14]; however, there are likely >1,000 hospitals that transfuse blood across Kenya [31]. Improving health outcomes across LMICs (e.g., reducing mortality due to obstetric haemorrhage) may require making blood available in distant transfusing facilities, with complex logistics related to collecting, testing, transporting and storage of blood that are included in the model.

Instead of using an outcome of increased blood supply like many previous studies, this study employs a patient-centric endpoint of the proportion of patient-level blood demand that is met, separated further along different patient types based on urgency of blood need. The selected metric directly reflects the effectiveness of the blood supply chain in meeting minimal patient needs and provides a clear indicator of the impact of different interventions on patient outcomes. The model also has the capability to use various outcomes, such as blood unit level metrics, depending on the unique needs or preferences of patients or decision-makers. This flexibility allows researchers, healthcare administrators and policymakers to tailor the analysis to specific needs and priorities, ensuring that the model’s insights are as relevant and actionable as possible.

There are two potential primary uses of this, or a similar, model for blood system stakeholders. First, the model could aid in understanding the sensitivity of the system to various input parameters that might not all be within direct control (e.g., the TTI pass rate, the mix of patients arriving at a particular hospital, or FRD response rates). This allows for a better understanding of potential impact or differences across contexts and would allow for more tailored allocation of human and material resources. Second, the model can be used to study the effects of planned or proposed interventions that could affect system behaviour (e.g., adding more blood drives, using faster modes of transportation for blood, sending blood samples for qualification testing more frequently, inventory management, storing and testing blood at the point of use, etc.). Many of these interventions could be evaluated with changes only to the input data; the goal of the team in future work is to minimise the need to modify the simulation logic, and ensuring it is easily adaptable for various sites across Kenya.

The three experiments presented here highlight the potential usefulness of this model approach. First, Experiment 1 demonstrates why consideration of demand-linked supply (FRDs are explicitly linked to patients with blood need) is important. The experiment describes comparable changes to two different strategies in collecting blood, one linked to demand and the other not. Both strategies target donors in the same community: either relatives of patients, or other members of the same community targeted by drives in town centres (institutional drives typically target student populations). Note that a 50% increase in either strategy, the actual percentage increase in blood collected and qualified will be significantly smaller than 50%. In our simulation, while doubling community drives nearly doubles donations from that channel, institutional drives and other donor sources remain unchanged, so the overall impact on blood availability is dampened. This underscores the effect of targeted interventions in a multi-source system and highlights why system-wide simulation is essential. Moreover, a 50% increase in either strategy (recruitment of FRDs per patient or number of blood drives) results in differing numbers of donated units appears to be materially different, demonstrating that the baseline blood drive frequency was likely already too low to support the demand: doubling community blood drives yields around 150 more qualified blood units annually, while doubling the FRD donation rate results in approximately 470 more units. While performance in the model is improved by maintaining a link between supply (blood donation) and demand (transfusion), future work will also need to compare costs and cost-effectiveness of either strategy. While the results do not advocate for any specific strategy in isolation, the experiment suggests that, based on the parameter set and assumptions in this model, strategies to maintain or increase demand-linked supply are important to consider. For instance, as recruitment strategies in the community that are connected to the patient in need may provide more incentive to donate, especially in smaller, remote hospitals where organising blood drives might be challenging due to not having available blood transfusion collection staff in the community or an outpost of the national transfusion service to organize such events. In the real world in Kenya however, FRDs in smaller, remote hospitals, likely face challenges to reach distant blood collection centres to donate blood. Future work will explore combinations of strategies where collection strategies in Beta hospitals are paired with structural changes to the blood system (e.g., local recruitment, collection and testing).

Experiment 2 demonstrates that the system is not very sensitive to variations in emergency vs non-emergency case mix or sporadic increases in emergency blood need. This is possibly due to the low overall met demand rate (30%), meaning that the system is experiencing a blood shortage regardless of demand fluctuations. While low met demand rate at baseline is reflected in quantitative data collected in the study (manuscript in preparation), we also ran all the experiments described here after artificially increasing blood supply. There were no qualitative differences to the findings presented here in a paradigm where baseline met demand rate is higher. When a patient is discharged before receiving their requested blood, their unused blood is reallocated to the next patient, and this rollover mechanism helps maintain stable fulfillment rates at the hospital level, even when the patient mix changes. This finding emphasises that the rate of meeting blood demand may be less variable from setting-to-setting based on case mix, and focus on other elements of the system may have a greater impact in informing potential future planning and interventions.

An interesting finding of the Experiment 3 was that a regular restock of blood to hospital labs could help improve meeting blood demand for patients in Beta hospitals without significantly compromising the overall met demand rate. Combining this restock strategy with reserving some blood for emergency use could significantly improve the met demand rate for emergency patients, while compromising care for non-emergency patients. The simulation therefore reveals the policy decisions that may be necessary, and their impact, in strategies that demonstrate great potential for ensuring a steady availability of blood for both routine and emergency needs, and reducing delays in critical situations. The simulation also reveals counterintuitive results like regular restocking might negatively impact performance in alpha hospitals (Fig 5) due to a combination of timing of when an order arrives at a shared, finite supply location, and additional hospitals performing regular restocks.

There are several limitations to this study. First, the results presented here are not meant to be definitive, nor are they meant to be prescriptive. Rather, the goal of this project is to provide illustrative examples of how a realistic, “vein-to-vein” simulation model of the blood system can be used to gain insights into a complex system, and how it can serve to evaluate various what-if scenarios and decision alternatives. We have validated the results wherever possible using data collected from hospitals. For example, 2,761 blood units were requested over a nine-month period at one Alpha hospital, while our simulation yielded a mean of 2,785.9 units (99% confidence interval: 2,759.47 to 2,812.27) over the same period across ten simulation runs. Similar data from Beta hospitals are not yet available, and additional validations will be conducted as new data become available. Some aspects of the blood continuum have been simplified for modelling purposes (e.g., lumping all patients into two broad categories based on urgency of blood need, assuming reasonable probability distributions for random factors, and focusing on blood as a single product while ignoring blood typing and specific blood components such as plasma or platelets). Therefore, further assessment, both within and outside of the model, must be conducted prior to implementing the results of these simulations into practice. Second, while the model presented here uses actual data wherever such data are available (e.g., on the demand side), and direct feedback from practitioners in the field where reliable data is unavailable, it does not perfectly reflect the reality of the entire system in every possible context. Thus, while the results presented are internally valid and externally informative, the specific estimated findings may not perfectly represent real-world results. Third, the model presented here is stochastic, in that it captures fluctuations in flows and events over time, but is not probabilistic, in that it does not reflect uncertainty in parameters such as mean daily events. This distinction is sometimes referred to as ‘first order’ versus ‘second order’ uncertainty [32]. Further development of the model to include second-order uncertainty would allow for probabilistic sensitivity analysis [33] that could provide additional value to policymakers by quantifying uncertainty around recommendations and identifying where further data collection would be most valuable. While the data required for probabilistic modelling of this blood continuum are not currently available, ongoing efforts to collect routine, real-time data on blood utilisation at multiple sites in Kenya [34], coupled with a realistic simulation model, open the possibility of creating a “digital twin” of the blood system where the operations of such complex health systems can be managed in more agile ways [35].

In conclusion, this paper presents the methods and illustrative results of a DES simulation model of the blood continuum in Kenya. By considering the entire blood continuum from “vein to vein,” the use of both quantitative and qualitative real-world data to inform model inputs, inclusion of a patient-centric outcome, and an incorporating diverse LMIC blood system contexts, this model provides a new and insightful approach to understanding and improving global health systems. Future research using this model, or incorporating approaches used in developing this model, could improve the ability of health system leaders to make operational, policy and funding decisions to increase the availability of safe blood for transfusion at the point-of-care across LMICs.

## Supporting information

S1 Fig The final comprehensive process map, illustrating the three stages of the blood continuum and colour coding key similarities and differences across 4 representative locations. (TIF)

S1 Checklist Inclusivity-in-global-research-questionnaire-PITS Kenya. (PDF)
